# P-1821. Prevalence and Risk Factors of Hepatitis B Virus Among Sexually Active Patients in Minna, Niger State: A Cross-Sectional Study

**DOI:** 10.1093/ofid/ofaf695.1990

**Published:** 2026-01-11

**Authors:** Aliyu Evuti Haruna, Nma Bida Alhaji, Isah Leje umar

**Affiliations:** Africa Center of Excellence for Mycotoxin and Food Safety, Federal University of Technology, Minna, Niger State, Minna, Niger, Nigeria; Africa Center of Excellence for Mycotoxin and Food Safety, Federal University of Technology, Minna, Niger State, Minna, Niger, Nigeria; 6Department of Medical Laboratory Science, General Hospital Minna, Niger State., Minna, Niger, Nigeria

## Abstract

**Background:**

Hepatitis B virus (HBV) remains a major public health concern, especially in developing countries where infection rates are significantly high. HBV can lead to serious complications including chronic liver disease, cirrhosis, and hepatocellular carcinoma. Understanding the socio-demographic and behavioral determinants of HBV transmission is essential for effective intervention. This study aimed to assess the prevalence of HBV and identify associated risk factors among sexually active patients attending General Hospital Minna, Niger State.

**Methods:**

A hospital-based cross-sectional study was conducted among 300 sexually active patients aged 15–65 years. Participants were selected randomly and data were collected using a self-administered questionnaire capturing demographic information, behavioral risk factors, and clinical history. Data were analyzed using SPSS version 24.0, employing descriptive statistics and chi-square tests to assess associations at a 0.05 level of significance.

**Results:**

The overall prevalence of HBV among participants was 12.7%. The majority of respondents (45%) were aged 26–35 years, and only 3% were older than 65 years. HBV prevalence was significantly associated with age, sex, marital status, and occupation (p < 0.05). Key risk factors identified included mouth-to-mouth kissing (17%), use of sharp instruments (14%), multiple sexual partners (8%), blood transfusions (14%), tribal marks (9%), surgical history (6%), alcoholism (5%), and family history of HBV (11%). A notable finding was that 42% of individuals with multiple sexual partners were full-time housewives.

**Conclusion:**

This study reports a relatively high HBV prevalence of 12.7% among sexually active individuals in Minna, Niger State. Demographic and behavioral factors significantly influenced infection risk. Targeted health education, HBV screening, and risk-reduction interventions are crucial in this population to reduce transmission and the associated disease burden.

**Disclosures:**

All Authors: No reported disclosures

